# Prioritizing gender equity and intersectionality in Canadian global health institutions and partnerships

**DOI:** 10.1371/journal.pgph.0001105

**Published:** 2022-10-04

**Authors:** Bianca Carducci, Emily C. Keats, Michelle Amri, Katrina M. Plamondon, Jeannie Shoveller, Onome Ako, F. Gigi Osler, Carol Henry, Nitika Pant Pai, Erica Di Ruggiero

**Affiliations:** 1 Department of International Health, Bloomberg School of Public Health, Johns Hopkins University, Baltimore, Maryland, United States of America; 2 Centre for Global Child Health, Hospital for Sick Children, Peter Gilgan Centre for Research, and Learning, Toronto, Ontario, Canada; 3 Takemi Program in International Health, Harvard T.H. Chan School of Public Health, Harvard University, Boston, Massachusetts, United States of America; 4 Faculty of Health and Social Development, School of Nursing, University of British Columbia, Kelowna, British Columbia, Canada; 5 Faculty of Medicine, Community Health and Epidemiology Department, Dalhousie University, Halifax, Nova Scotia, Canada; 6 IWH Health Centre, Halifax, Nova Scotia, Canada; 7 Action Against Hunger, Toronto, Ontario, Canada; 8 Canadian Partnership for Women and Children’s Health (CanWaCH), Peterborough, Canada; 9 Department of Otolaryngology-Head and Neck Surgery, University of Manitoba, Winnipeg, Manitoba, Canada; 10 Federation of Medical Women of Canada, Ottawa, Ontario, Canada; 11 Division of Nutrition, College of Pharmacy and Nutrition, University of Saskatchewan, Saskatoon, Saskatchewan, Canada; 12 Department of Medicine, McGill University, Montreal, Quebec, Canada; 13 Social and Behavioural Health Sciences Division, Dalla Lana School of Public Health, University of Toronto, Toronto, Ontario, Canada; 14 Centre for Global Health, Dalla Lana School of Public Health, University of Toronto, Toronto, Ontario, Canada; 15 Institute of Health Policy, Management and Evaluation, Dalla Lana School of Public Health, University of Toronto, Toronto, Ontario, Canada; University of Zambia School of Public Health, ZAMBIA

## Abstract

Despite governmental efforts to close the gender gap and global calls including Sustainable Development Goal 5 to promote gender equality, the sobering reality is that gender inequities continue to persist in Canadian global health institutions. Moreover, from health to the economy, security to social protection, COVID-19 has exposed and heightened pre-existing inequities, with women, especially marginalized women, being disproportionately impacted. Women, particularly women who face bias along multiple identity dimensions, continue to be at risk of being excluded or delegitimized as participants in the global health workforce and continue to face barriers in career advancement to leadership, management and governance positions in Canada. These inequities have downstream effects on the policies and programmes, including global health efforts intended to support equitable partnerships with colleagues in low- and middle- income countries. We review current institutional gender inequities in Canadian global health research, policy and practice and by extension, our global partnerships. Informed by this review, we offer four priority actions for institutional leaders and managers to gender-transform Canadian global health institutions to accompany both the immediate response and longer-term recovery efforts of COVID-19. In particular, we call for the need for tracking indicators of gender parity within and across our institutions and in global health research (e.g., representation and participation, pay, promotions, training opportunities, unpaid care work), accountability and progressive action.

## Introduction

In a recent investigative series, a Canadian newspaper, the Globe and Mail, highlighted a *Power Gap* [1] between women and men in the Canadian workforce. Their unprecedented analysis of 82 universities revealed that women are outnumbered, outranked and outpaid in the highest decision-making and leadership positions [1]. Compounding this, emerging evidence suggests that the severe acute respiratory syndrome coronavirus 2 (SARS Cov-2) and its mitigation responses (together hereinafter referred to as COVID-19) have perpetuated pre-existing gender-based inequities and disproportionately impacted those already adversely affected by other social determinants of health [2]. Impacts including but not limited to, greater risk of exposure to COVID-19, employment and income losses, increased care demands, increased gender-based violence, and unequal access to education and technology, are creating a systemic human development crisis [2].

Explicit attention to these gendered impacts is directly aligned with Sustainable Development Goal (SDG) 5 calling on all nations including Canada to achieve gender equality not only because it is a fundamental human right but a “necessary foundation for a peaceful, prosperous and sustainable world” [3]. Furthermore, Canada’s approach to planning, including any COVID-19 recovery plans should consider both the direct and indirect impacts on gender and its intersections with other social identities, while also promoting inclusive growth and sustainable development outcomes [4]. It is therefore critical that all Canadian organizations, including those working in global health contribute to this call to action to promote gender equality.

Gender refers to the non-binary social construct of characteristics, experiences and expressions of individuals, inter alia, the manifestation and evolution of roles, behaviours, and attributes [5]. However, beyond gender, multiple systems of power intersect to generate systemic discrimination and structures of privilege that impact individuals and societies. In other words, one must also consider the intersections of gender [6]. In her 1989 paper, Kimberlé Crenshaw defined intersectionality as a lens to understand how aspects of an individual’s social and political identities (e.g., gender, sex, caste, class, religion, disability, physical appearance, sexual orientation) when combined, create different axes of systemic discrimination and privilege [7]. With regards to health outcomes and its extensions, especially in the context of COVID-19, evidence reveals a disproportionate risk of communicable and non-communicable diseases is due to structural inequities that fall at the intersections of unearned advantage (and disadvantage) associated with gender, race, ethnicity, and class [8].

Efforts to promote gender equity in Canada involve a patchwork of approaches including human rights-based policies (e.g., Royal Commission on the Status of Women in Canada; Canadian Charter of Rights and Freedoms) [9]. In 2021, the International Development Research Centre released its 10-year strategy renewing its commitment to gender equality [10] and the Canadian Institutes of Health Research (CIHR)’s strategic plan included a strong and unprecedented focus on the social determinants of health, and on health equity (including gender equity) [11]. These tenets were also reflected in CIHR’s new Global Health Framework [12]. Canada has also actively participated in several international platforms to support gender equality. In 2017, Canada’s Feminist International Assistance Policy (FIAP) was adopted to forward the agenda of gender equality and empowerment of women and girls and their human rights, as part of its international aid efforts and engagement in global health work [13,14]. However, FIAP is not without shortcomings, and there is growing concern that Canada is not doing enough to address women’s rights in Canadian global health institutions domestically or within our partnerships abroad [15]. According to Cadesky (2020), the FIAP simultaneously over-politicizes and depoliticizes feminism, gender, and gender equality to suit prevailing environments, while shifting the spotlight away from structural issues of social and economic justice that contribute to gender inequities [15]. Moreover, the absence of decolonial feminism theory within national policies further threatens Canada’s progress on SDG 5 and international relations efforts.

In the Canadian context, global health is rooted in and influenced by ongoing effects of colonialism; though there are concerted efforts to decolonize global health research [16–19]. This narrative review examines institutional gender inequities and inequalities in Canadian global health research, policies and practices. We recommend four priority actions for Canadian institutions to transform gender equality and equity within the workplace (Actions 1–3) and their work internationally (Action 4), in the wake of COVID-19.

## Methods

We conducted a narrative review to summarize existing literature, illustrate points of debate and highlight knowledge gaps on gender equity in Canadian global health. To inform our search strategy, we performed an initial review of grey literature including institutional documents and websites related to gender equity in global health purposefully identified by project team members. We then developed a search strategy for MEDLINE (Table 1) using keywords, limiting results to the Canadian context and English language articles published after the year 2000 (Search date: July 22, 2022). Titles and abstracts were reviewed to capture empirical or conceptual studies related to gender, institutional domains and activities in the Canadian global health ecosystem. After eliminating duplicates, we included 16 articles. From these articles and grey literature, we derived actions related to current data and policies, representation in leadership and governance, and workplace conditions. This study did not receive nor require ethics approval, as it does not involve human and or animal participants.

**Table 1:** Search strategy.

Concept 1—Gender	Concept 2—Institutional Domains	Concept 3—Institutional Activities	Concept 4—Global Health
Gender	Research	Leadership	Global Health
Equity, Diversity, Inclusion	Policy	Representation	International Health
Gender equity	Capacity Building	Governance	
Gender equality	Education	Mentorship	
Intersectionality	Practice	Partnership	
Women	Advocacy	Training	

For the purposes of this paper, we define Canadian global health institutions as Canadian-based public or private sector organizations. This includes Canadian-based multilateral and bilateral development institutions, non-governmental organizations and civil society partners, funding bodies, higher-education or research institutes which advance global health research, policy, and practice globally (Panel 1). We further draw attention to Canadian-based women leading global health research, policy, and practice initiatives internationally. In doing so, we also recognize that disparities are amplified for gender non-conforming people and people navigating multiple intersecting forms of social exclusion (e.g., racism; ableism; ageism; classism).

## Navigating power dynamics in Canadian global health institutions

Progress on SDG 5 is sluggish globally [20]. As the impact of the COVID-19 pandemic continues to be felt, closing the global gender gap has increased by a generation from 100 years to 132 years, according to the 2022 World Economic Forum Global Gender Gap Index, which benchmarks 156 countries on global parity [21]. Canada ranks above the global average at 25^th^; however, Canada has fallen six spots since 2020– primarily due to poor performance in two of four subindices: 1) political empowerment (from 25^th^ to 31^st^) and 2) economic participation and opportunity (from 30^th^ to 43^rd^); health and survival and educational attainment indices have remained stable [21]. Metrics reveal widening gaps in participation, advancement and remuneration between men and women, related to the “glass ceiling” and “glass cliff” (Panel 1) [21]. Despite gender parity in Canada’s COVID-19 task force [22], there is no shortage of examples of both phenomena, especially with regards to Canadian women’s economic participation and political empowerment in global health leadership [23]. Several women are the face of public health measures in Canada to control COVID-19 at the national, provincial, and local level. While they have been praised for their exceptional leadership, they have also been the subject of criticism, harassment, and racial discrimination [24,25].

Within the academy, the lack of gender parity in Canadian-led global health research in terms of production of global health knowledge and representation on editorial boards is apparent [26]. A review of over 23,000 CIHR grant applications between 2011 to 2016 concluded that grants led by women principal investigators were evaluated less favourably, despite similar quality of proposals led by men and women [27]. Likewise, an analysis of *Lancet Global Health* publications (between June 2013 to Nov 2018), indicated women constituted only 34% of all Canadian authorship lists. A key caveat is the assumption of gender with regards to authors (i.e., those who self-identify as women), as most journals do not require gender declarations upon submission. This proportion was significantly lower for women in low- and middle-income countries [28]. Further, numerous examinations into scholarly publishing during COVID-19 suggests a substantial decline in authorship for women, but an increase for men; highlighting additional barriers to academic career advancement that are bound up in women’s multiple roles in the household [29].

Socio-structural factors create workplace demands and cultures which tend to reflect life patterns and gender roles of men. Such patterns are often at odds with the time and resource constraints that women usually face as primary caregivers. The disproportionate burden of unpaid care work for family [30], combined with structural disadvantages associated with having children (or “child penalty”) and limited support systems such as accessible day care, all contribute to women’s cumulative loss in lifetime earnings, productivity and opportunities [31]. Despite accounting for the majority of the global health social workforce [32], the lack of social protections and job security for Canadian women, especially Black, Indigenous and Persons of Colour (BIPOC) women, is persistent for those working in global health, especially internationally-based global health practice and humanitarian aid and relief organizations [33]. This situation is further aggravated by the lack of publicly available data on pay transparency and the gender wage gap, with Canada ranking among the worst Organisation for Economic Cooperation and Development countries in 2020 [34,35]. Importantly, these data do not accurately capture the disparities which may exist in disaggregated data and the vast number of women in precarious part-time or temporary jobs, which were largely lost during COVID-19 [30]. Despite Canada’s values and aspirations to advance a ‘feminist agenda’, the lack of collective action to dismantle the layers of colonialism and systemic advantage afforded to men, continue to perpetuate the *Power Gap* that disadvantages women.

## Mentorship and resources to support empowerment and advancement of Canadian women in global health

The need for mentorship of women is particularly evident when reflecting on a potential disconnect between Canadian women training in global health and rising to leadership positions [36], especially for BIPOC women. Canadian academic institutions, non-governmental organizations, and professional health societies and associations are well-positioned to lead by example and contribute to the elimination of harmful practices that perpetuate gender and intersectional inequities. Universities educate and prepare the next generation of leaders for roles in global health research, policy, and practice, while non-governmental organizations and health societies and associations are an excellent conduit for continuing education (i.e., practicums and internships), networking and advocacy [37]. However, the vast field of global health encompasses a wide array of disciplines and expertise—ranging from community-based research, epidemiology program evaluation, policy development, and many others—making it difficult for one organization, school or program to provide comprehensive training to well-equip young leaders for a plethora of potential career paths or projects in the field [37]. Additionally, few existing Canadian university programs or Canadian-based global health mentorship programs explicitly focus their efforts on the unique circumstances of *women*.

To meet a mentorship gap, the Canadian Association for Global Health (CAGH), formerly Canadian Society for International Health, created a formal mentor program, MentorNet [38]. While programs like MentorNet have demonstrated success both at the individual-level (e.g. promoting learning, development, networking, and others) and more broadly (e.g. through contributing to education of a wider array of global health topics and values that are not always communicated in a classroom), the program is not focused on women and only has the capacity to match approximately 30 pairs per year (despite receiving over three- or four-fold this number in applications) [38]. Increased investment in mentorship and sponsorship, especially for women early in their careers could enable more women to transition from their graduate studies and entry-level positions through to mid-career in global health research, policy and practice realms [39].

## Canada’s platform for gender-sensitive partnerships and standardizing global gender data and intersectional approaches in research

Gender-related gaps in data manifest in global health research, for example, in data collection, analysis and dissemination and they also exist at the institutional level, in terms of research team composition in Canada and our partnerships globally [4]. In both cases, the lack of gender-disaggregated data and empirical accounts of women’s lived experiences, particularly relating to gender and its intersections, make identifying large-scale issues and tracking change over time more challenging. Contributing to this issue, is the lack of requirement by most journals and funders to mandate disaggregated data by gender, where applicable, or ask for evidence of a gender mainstreaming approach to grantees’ work [40]. The absence of data has become particularly problematic in the context of COVID-19, given its disproportionate toll on women in terms of health and nutrition, loss of education and economic opportunities, experiences of interpersonal violence, and the need to plan national-level responses that take gender and its intersections into account [2,41]. Within Canadian global health research, a lack of disaggregated data means that programmes cannot be as gender-responsive as they should be [4,5].

The Canadian federal government (Global Affairs Canada) created the Department of Women and Gender Equality and national committees and forums, which developed guidance and tools (Gender-based Analysis Plus), and, mandated “no less than 95 percent of Canada’s bilateral international development assistance initiatives will target or integrate gender equality and the empowerment of women and girls” [13]. Moreover, CIHR launched the Institute for Gender and Health to foster research that explores how sex and gender influence health [42]. These actions have yet to result in changed institutional culture and there is limited monitoring and evaluation of gender within Canadian global health leadership and partnerships in terms of gender equity. This impacts our understanding of who leads or forms global health partnerships, and what their roles and opportunities are in those partnerships, including the co-creation of global health research [4,5].

Importantly, the relational skills (e.g., time, trust-building, responsiveness) needed to cultivate collaborative partnerships are gendered and de-valued in tenure, awards, or promotion [43,44] compared to more individually-driven and competitive academic outputs [45,46]. Women in Canadian academic institutions also tend to invest in relational and collaborative ways of working, including building partnerships and informal mentoring that are not always captured by traditional metrics of productivity [43]. There is a role within Canadian institutions for ensuring transformative change relating to partnership building through decolonization [47,48].

## Priority actions to gender-transform Canadian global health institutions

Despite governmental commitments to gender equality, Canadian women in global health are underrepresented and underpaid in leadership, management and governance positions in the public and private sector. The coming years present a window of opportunity to decolonize global health through a feminist lens, where a paradigm, leadership and knowledge shift are necessary across Canadian global health institutions to make women and their intersections count. We propose four priority actions to gender-transform Canadian global health institutions (Panel 2).

### Promote the development and monitoring of gender within equity, diversity and inclusion strategies in Canadian global health institutions

When women are treated unequally or do not receive the same opportunities as men, workplaces are less progressive, innovative, and effective [49]. Canadian global health institutions need to move beyond rhetoric and address systemic structural inequities (in management, leadership and governance positions, as well as in global health partnerships) [50,51]. Changing institutional culture requires meaningful Equity, Diversity and Inclusion (EDI) strategies that move beyond performative allyship [51]. As a start, Canadian global health institutions have a responsibility to promote the visibility of women’s contributions and normalize diversity [51] by building on existing momentum such as the CAGH Women in Global Health and using Athena Scientific Women’s Academic Network Charter as institutional guidance [52,53]. For example, moving beyond the overreliance of traditional performance indicators (i.e., “publish or perish”) is crucial, especially when hiring, promoting, awarding, and tenuring in global health academic settings. Rewarding a range of contributions (e.g., informal mentorship of students and colleagues, beyond the number of students supervised) in merit and promotion/tenure processes is essential.

### Achieve gender parity in Canadian global health management, leadership and governance roles and collaborative research teams through mentorship and sponsorship

It is imperative for Canadian global health institutions to implement transformative leadership models and enabling environments, whereby gender parity can be achieved in global health research, practice, and policy domains. Strengthening mentorship programs, expanding practice opportunities and leadership and management curricula are critical to supporting and increasing the visibility and recognition of women in global health research and policy. Initiatives, such as Stanford WomenLift Health [54] have the potential to accelerate the advancement of mid-career women’s talent to leadership, catalyze organizational change, and rebuild movement towards gender parity in global health research and policy. In implementing such initiatives, it is crucial to ensure that mentorship of women does not solely fall on the responsibility of women, which is often the case, and that this work is made visible and rewarded, including in career advancement. While gender-sensitive mentorship is necessary, it is insufficient to challenge social norms and redress systemic inequities in career paths for women [55].

### Champion safe and flexible workplaces which value women’s work and ensure equitable employment conditions

The International Labour Organization calls for the further development and monitoring of gender-sensitive workplace policies [56]. Such policies can illuminate the differences that exist between women and men workers and help identify and address differential physical, psychosocial, safety and health risks [56]. The World Health Organization has developed a guide for employers and work representatives, which includes tools that “promote healthy and equitable workplaces for women and men” while drawing particular attention to health, economic and social issues predominantly affecting women” [57].

As a middle power, Canada occupies a position of influence on the world stage, and for decades has used this to advance agendas that purportedly prioritize equity, human rights, and the well-being and status of women [13–15]. Institutional commitments including workplace policies need to be re-examined and strengthened to better attend to safety and flexibility concerns in an effort to improve gender equality. Global health institutions have long been plagued with sexism and reflect a profound hierarchy that reinforces harmful practices and inadvertent behaviours that can disadvantage women [58]. All workers, including leadership, management, and governance teams in global health should be required to undergo gender equality training (i.e., cultural sensitivity, and self-awareness of implicit bias and gender stereotypes), and the fulsome integration into scientific and medical curricula of a comprehensive approach to EDI (e.g., not a “one-off” session) should be standard practice [51]. Galvanizing collective commitment, solidarity and practicing critical allyship [59] will help combat unconscious bias and ensure authentic inclusion of diverse Canadian women in global health [60].

Furthermore, to curb the further marginalization of women and their intersections, institutional policies need to be designed to promote greater transparency by global health organizations (and other workplaces) on matters such as wage gaps between men and women employed in global health research and policy positions [61]. In an academic context, programs that assist women scientists in pivoting their research while juggling other care responsibilities outside of work should be enacted (e.g., institutional supports such as mentorship and bridge grants). For such policies to be effective for all workers, they must be informed by better data on the relationship between gender and work, and their implications for health and gender equality in the Canadian context.

### Collect and report on gendered data in Canadian global health research

Visibility and power inequities have a cyclical relationship: without attention and awareness, inequities flourish and gendered assumptions are left unquestioned. We urge those working in Canadian-led global health research to examine sex and gender as constructs and variables in the development of research questions, methodologies, plans for analysis and knowledge translation to action, in order to systematically and explicitly study gender inequities [62]. This includes non-governmental organizations, which play a pivotal role not only in the production of knowledge, but also in capacity building, resource mobilization, sharing and utilization of research findings, and networking [63]. For example, the use of a feminist approach could guide the development of research questions that are inclusive of the multiple vantage points in global health and self-reflexive about potential exclusions, including power and politics in all places within and beyond the conventional boundaries of states and international public spheres. In addition, it is critical to ensure the collection of gender-differentiated and equity data within global health research processes, programmatic implementation, and partnerships such as grantee selection, team and partnership structure (i.e., division of labour and decision-making) [52]. There has been a proliferation of proposed gender equality tools [64], equity principles and tools for partnerships [65,66] and empowerment-related indicators [67,68], including the Canadian-developed GENDER Index [69], but data collection methods and conceptual shortfalls have substantially limited their use. Collecting and reporting of data on equal partnerships is one way to monitor SDG progress [70], and these data should be publicly reported for accountability purposes and ongoing learning.

Finally, drawing on insights from the Gender and COVID-19 working group, gender-responsive pandemic planning needs to be data-driven and include the ethical collection of intersectional disaggregated data. This will ensure evidence-informed stewardship such that key areas which affect women and their intersections are financially and materially resourced [71]. Further, engagement and collaboration of global health stakeholders in COVID-19 decision-making in national, bilateral and other fora (e.g., World Health Assembly) should include women, their intersections and equity-seeking individuals, civil society, and global health partners.

### Making a difference

Canada’s role in international affairs is inextricably linked to its history of colonization [14]. Advancing gender equity within Canadian institutions for global health is a vital step to becoming more equitable, diverse and power-symmetric [18,19,72]. We must create opportunities, retain diverse Canadian talent, and enlist our partners to do the same for a resilient and dignified future of work. Doing so will reap a multiplier effect in health, empowerment, and economic and social opportunities, or a “triple gender dividend” [32]. With less than 10 years left to achieve the SDGs, Canada needs to reimagine its role in creating futures that dismantle colonial and patriarchal structures that entrench gender inequities.

### Panel 1: Definitions

**Gender equality** is defined as women and men who enjoy the same status and have equal opportunity to realize their full human rights and potential to contribute to national, political, economic, social and cultural development, and to benefit from the results [13].

**Gender equity** is defined by Global Affairs Canada as being fair to women and men to compensate for social and historical disadvantages that prevent women and men from otherwise operating as equals [13]. In the context of health, while inequality is measurable, inequity is a political concept with a commitment to social justice [73]. In other words, while inequalities are believed to be unnecessary and avoidable, inequities are understood as also being unfair and unjust [74,75].

**Gender mainstreaming** is the process of assessing the implications for women and men of any planned action, including legislation, policies, or programmes, in all areas and at all levels [40].

**Gender non-conforming** is an umbrella term referring to people who do not identify in a way that conforms to the traditional expectations of their gender, or whose gender expression does not fit neatly into a category. Some gender non-conforming people identify as non-binary, genderqueer, trans masculine, trans feminine, agender, bigender, or other identities that reflect their personal experience [76].

**Gender parity** concerns relative equality in terms of numbers and proportions of women and men, girls and boys, and is often calculated as the ratio of female-to-male values for a given indicator.

**Gender wage gap** is defined as the difference between median earnings of men and women relative to median earnings of men [34].

**Glass ceiling or Glass wall** results in gender segregation in management functions, limiting the talent pool of women that institutions can tap into for candidates to fill top executive and CEO positions [48].

**Glass cliff** is a silent phenomenon where women and minorities are appointed to precarious top positions during times of crises, where there is a high probability of failure [25].

**Global health institution** is defined as public or private organizations (i.e., multilateral, bilateral, non-governmental organizations and civil society partners, funding bodies, higher-education or research institutes) which advance global health research, policy and practice globally. For the purposes of this paper, Canadian global health institutions are those which have a Canadian headquarters or branch.

**Intersectionality** moves beyond examining individual factors such as biology, socioeconomic status, sex, gender, and race. Instead, it focuses on the relationships and interactions between such factors, and across multiple levels of society, to determine how health is shaped across population groups and geographical contexts [6].

### Panel 2: Priority actions for Canadian global health institutions

#### Action 1: Promote the development and monitoring of gender equity, diversity and inclusion strategies in Canadian global health institutions

1. Adapt the Athena Scientific Women’s Academic Network Charter to ensure gender equality is prioritized throughout research, practice, capacity building and advocacy portfolios, while recognizing Canada’s multicultural and Indigenous diversity
2. Establish procedures and set gender budget targets, including funding gender-specific academic and clinician roles
3. Conduct rigorous and transparent institutional gender assessments to benchmark annual progress and implement gender-transformative actions
4. Ensure representation of women, their intersections and equity-seeking individuals in institutional COVID-19 response planning and decision-making

#### Action 2: Achieve gender parity in Canadian global health management, leadership and governance roles and collaborative research teams through mentorship and sponsorship

1. Identify leadership and partnership models which proactively promote gender-transformative pathways for recruitment and retainment within Canadian global health institutions
2. Build a social movement of change to empower and support the growth of early and mid-career women in global health through formalized career and skills development and mentorship at scale
3. Strengthen ongoing efforts by Canadian Association for Global Health to build a database of Canadian women in global health to promote visibility, opportunities for networking and track talent

#### Action 3: Champion safe and flexible workplaces which value women’s work and ensure equitable employment conditions

1. Create a culture that normalizes dual roles of Canadian women and values women’s unpaid care work by reforming workplace policies which support flexible work arrangements, work-life balance and safe workplace conditions
2. Redress gender pay and promotion gaps through pay transparency
3. Facilitate and advocate for allyship and solidarity through gender bias training

#### Action 4: Collect and report gendered data in Canadian global health research

1. Support and track partnerships between Canadian institutions and global health institutions that mainstream gender throughout a project life cycle, including prioritizing the co-creation of research with women from the Global South
2. Mandate analyses of sex-, and where possible gender-, disaggregated data that includes other stratifiers of social and health equity (e.g., race, sexuality, religion) in Canadian global health research
3. Promote voluntary disclosure of gender and its intersections during manuscript submission to monitor authorship inequities
4. Advocate for funding bodies to disclose funding success rates by gender with the intention to advance equity in Canada
5. Ensure Principles for Global Health Research are applied in data mining and evidence synthesis exercises in the context of gender, its intersections and COVID-19
